# Metformin enhances the radiosensitivity of human liver cancer cells to γ–rays and carbon ion beams

**DOI:** 10.18632/oncotarget.12966

**Published:** 2016-10-27

**Authors:** Eun Ho Kim, Mi-Sook Kim, Yoshiya Furusawa, Akiko Uzawa, Soorim Han, Won-Gyun Jung, Sei Sai

**Affiliations:** ^1^ Division of Heavy Ion Clinical Research, Korea Institute of Radiological and Medical Sciences, Gongneung-dong, Nowon-Gu, Seoul, South Korea; ^2^ Department of Radiation Oncology, Korea Institute of Radiological and Medical Sciences, Seoul, South Korea; ^3^ Department of Basic Medical Sciences for Radiation Damages, National Institute of Radiological Sciences, National Institutes for Quantum and Radiological Science and Technology, Chiba, Japan

**Keywords:** metformin, carbon ion beam, radiosensitivity, hepatocellular carcinoma cell, DNA damage

## Abstract

The purpose of this study was to investigate the effect of metformin on the responses of hepatocellular carcinoma (HCC) cells to γ–rays (low-linear energy transfer (LET) radiation) and carbon-ion beams (high-LET radiation). HCC cells were pretreated with metformin and exposed to a single dose of γ–rays or carbon ion beams. Metformin treatment increased radiation-induced clonogenic cell death, DNA damage, and apoptosis. Carbon ion beams combined with metformin were more effective than carbon ion beams or γ-rays alone at inducing subG1 and decreasing G2/M arrest, reducing the expression of vimentin, enhancing phospho-AMPK expression, and suppressing phospho-mTOR and phospho-Akt. Thus, metformin effectively enhanced the therapeutic effect of radiation with a wide range of LET, in particular carbon ion beams and it may be useful for increasing the clinical efficacy of carbon ion beams.

## INTRODUCTION

Hepatocellular carcinoma (HCC) accounts for 90% of all liver cancers, with approximately 80% of HCC cases occurring in the Asia-Pacific region [1, 2]. Despite considerable improvements in techniques, complete surgical resection is possible in less than 20% of HCCs. Furthermore, conventional chemotherapy and radiotherapy do not significantly benefit patients with advanced HCC [3, 4].

High linear energy transfer (High-LET) particle therapy produces spread out Bragg's peaks (SOBP), allowing it to cover tumors with biologically equivalent dose distributions. High-LET radiation has several advantages over low-LET radiation in treating radioresistant human cancers because of its higher relative biological effectiveness (RBE), lower oxygen enhancement ratio (OER), and decreased cell-cycle-dependent radiosensitivity. In addition, because cells are less likely to repair radiation injury, charged particle radiation may exert highly lethal effects even on radioresistant tumors, compared to conventional low-LET X-ray or γ-ray irradiation [5–8].

The Heavy-Ion Medical Accellerator in Chiba (HIMAC) is the first heavy-ion accelerator dedicated to medicine, and it has become the world's leading heavy-ion cancer treatment facility. Over the past 20 years, HIMAC has treated more than 500 HCC patients and achieved similar or better results than surgery. Because heavy ion radiotherapy is applied in only two fractions, the patient's quality of life (QOL) is also markedly improved [9, 10]. However, carbon ion radiotherapy alone has some limitations in the treatment of advanced tumors, particularly those located near important organs, and in the treatment of patients with micrometastasis [11, 12]. A recent clinical trial of carbon ion beams combined with gemcitabine achieved promising results for locally advanced pancreatic cancer, with 54% patients achieving 2-year overall survival [13]. We previously investigated whether the elimination of pancreatic cancer stem cells (CSCs) accounted for the high tumor control in that study [14].

Metformin (1,1-dimethylbiguanide hydrochloride), the most widely used treatment for type 2 diabetes, has recently sparked interest as a potential anticancer agent [15]. Metformin provides a synergistic benefit with chemotherapy or radiotherapy against certain cancers [15–23]. We have previously reported that metformin has radiosensitizing effects on HCC cells treated with high-LET neutron irradiation [24]. However, its application is greatly limited, with only three centers, worldwide, currently treating cancer using fast neutrons. This is perhaps because of a lack of funding and/or issues related to regulatory approval. An alternative approach may be to use other forms of high-LET radiation, such as carbon beams, which are widely used in Japan and Europe. In this study, we investigated the potential effects of metformin to sensitize HCC cells to carbon ion beams.

## RESULTS

### Clonogenic survival of Huh7 and HepG2 cells by γ-ray or carbon ion beams alone or in combination with metformin

**Figure 1:**
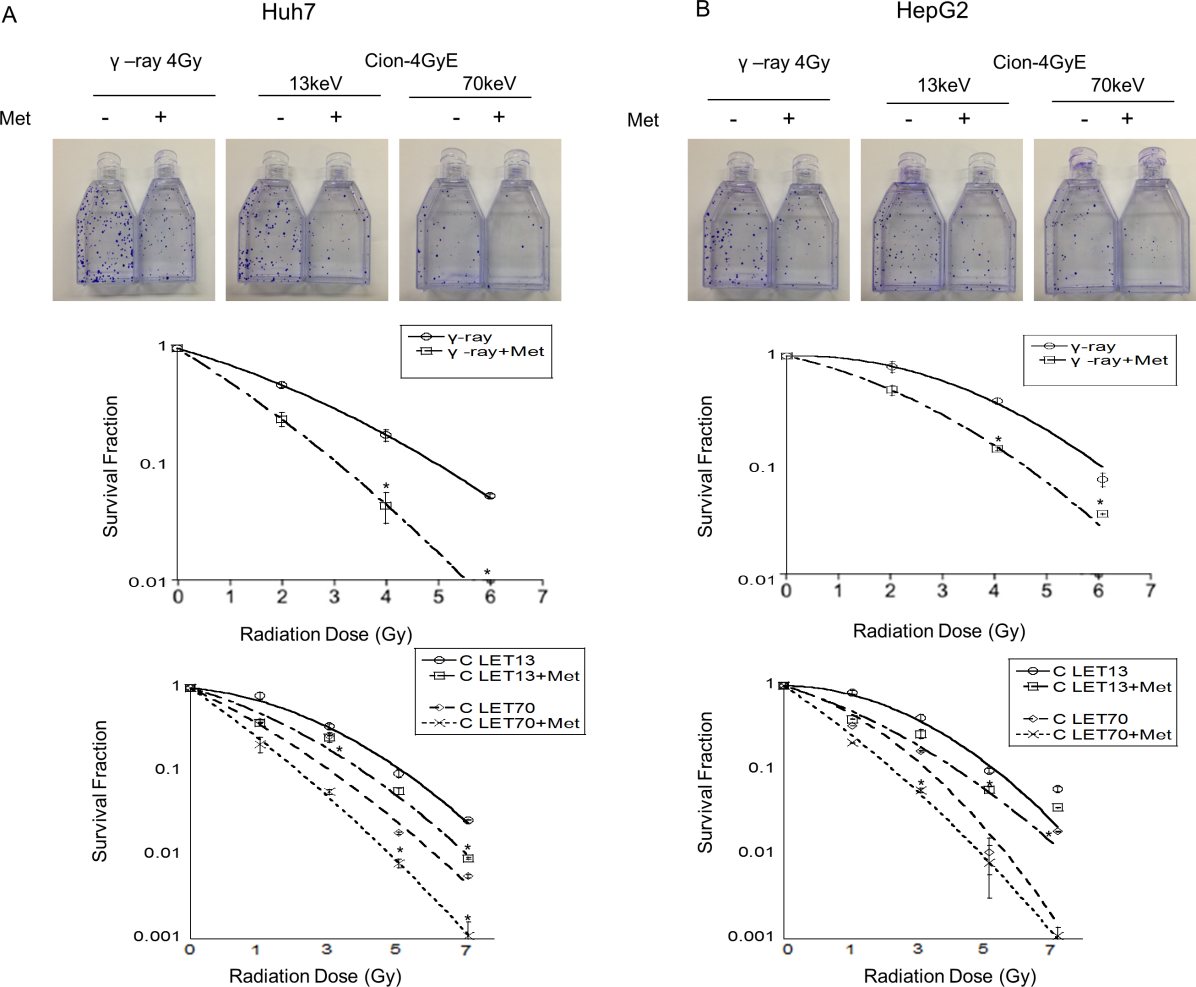
(**A**, **B**) Surviving fraction of Huh7 and HepG2 cells with and without metformin after γ-ray and carbon radiation. Huh7 and HepG2 cells were plated immediately after carbon ion beam and γ -ray irradiation alone or in combination with metformin (Met, 5 mM) for 48 h. The graphs show the mean +/− SEM calculated from 3 independent experiments.

Figure 1 shows that the clonogenic survival of Huh7 and HepG2 HCC cells decreased as a function of radiation dose after exposure to γ-ray or carbon ion beams (13 and 70 keV/μm). The average D10 (radiation dose for reducing cell survival to 10%) of Huh7 (HepG2) was 5.2 (5.6) Gy for γ-rays, whereas it was 5.1 (5.2) Gy and 3.1 (3.2) Gy for 13 and 70 keV/μm carbon ion beams, respectively (Table 1). Therefore, the RBE values of carbon ion beams with 13 and 70 keV/μm relative to γ-ray were 1.02 (1.08) and 1.68 (1.75), respectively (Table 1). The radiosensitizing effect of metformin was studied using 5 mM metformin, the dose that reduced cell survival by 25% with a 48 h incubation (data not shown). The D10 of Huh7 (HepG2) for γ-rays combined with metformin was 3.1 (4.6) Gy, whereas the D10s for 13 and 70 keV/μm carbon ion beams were 4.0 (3.9) Gy and 1.8 (2.0) Gy, respectively (Table 1). The radiosensitivity enhancement factor values and dose reduction for IR treatment of metformin -pretreated HCC cells are shown (Table 2).

**Table 1:** RBE values at D10 level for Huh7 and HepG2 cells after γ-ray, carbon ion alone or in combination with metformin

Cell Type	γ-ray	C-ion		RBE	
		13kev	70kev	13kev	70kev
Huh7	5.2Gy	5.1Gy	3.1Gy	1.02	1.68
+metfromin	3.1 Gy	4.0Gy	1.8 Gy		
HepG2	5.6Gy	5.2Gy	3.2Gy	1.08	1.75
+ metformin	4.6Gy	3.9Gy	2.0Gy		

**Table 2:** Radiosensitivity enhancement factor (REF) and dose reduction values

Cell type	Radiation type	REF value	Dose reduction (%)
Huh7	γ-ray	1.68	40.4
	C-ion 13keV	1.28	21.6
	C-ion 70keV	1.72	41.9
HepG2	γ-ray	1.22	17.9
	C-ion 13keV	1.33	25.0
	C-ion 70keV	1.60	37.5

The radiosensitivity enhancement factor (REF) corresponding to 90% cell killing was calculated to quantify the radiosensitization due to metformin using the equation. REF = radiation dose for 90% killing/radiation dose in the presence of metformin for 90% killing.

### Induction of DNA damage by γ-ray and carbon ion beams alone or in combination with metformin

**Figure 2:**
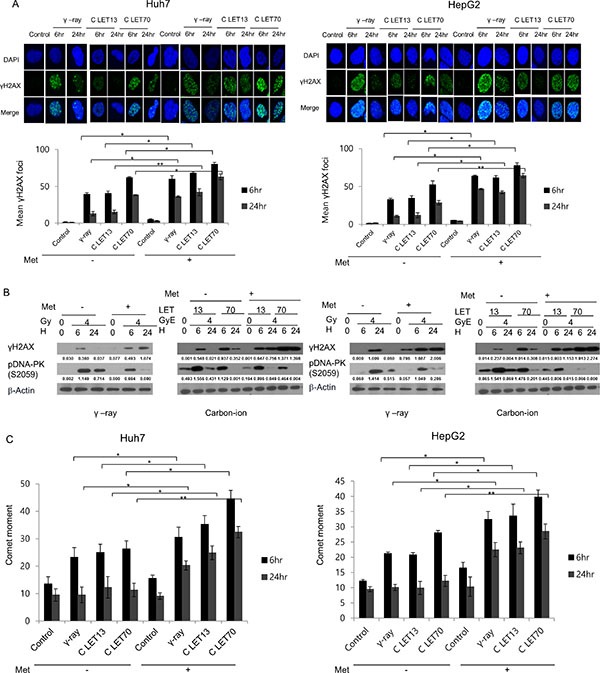
(**A**) Immunofluorescence staining for γH2AX phosphorylation in Huh7 and HepG2 cells after γ-ray and carbon ion beam alone or in combination with metformin (Met, 5 mM) at 6 and 24 h. (**B**) Cell lysates were immunoblotted with the indicated antibodies. (**C**) Neutral comet assay in Huh7 and HepG2 cells after γ-rays and carbon ion beams alone or in combination with metformin (Met, 5 mM) at 6 and 24 h. Values represent the means +/− SD of 3 experiments; *p < 0.05, **p < 0.001.

To elucidate the involvement of DNA damage in metformin-induced radiosensitization, we analyzed the formation of γH2AX. The number of γH2AX foci was initially increased 6 h after irradiation with either 4 Gy γ-ray or 4 GyE carbon ion beams, but then substantially declined after 24 h. Metformin treatment alone only slightly increased γH2AX expression, but markedly increased the effects of γ-rays and carbon ion beams on γH2AX (Figure 2). Notably, γH2AX foci formation by 4 GyE carbon ion irradiation was similar to, or slightly greater than, that caused by 4 Gy γ-ray irradiation either with or without metformin treatment.

Western blot results for γH2AX expression (Figure 2B) were in agreement with the results of γH2AX foci formation (Figure 2A). The expression of γH2AX upon irradiation with either 4 Gy γ -rays of 4 GyE carbon beams was enhanced by metformin. As shown in Figure 2B, irradiation with γ -rays or carbon ion beams increased the expression of phospho-DNA-PK, and metformin suppressed the radiation-induced increase in phospho-DNA-PK. The results of the comet assay indicated that metformin alone caused only slight DNA damage, while combined treatment further increased significant DNA damage, as indicated by an increase in comet movement (Figure 2C).

### Apoptosis is induced by γ-rays and carbon ion beams alone or in combination with metformin

**Figure 3:**
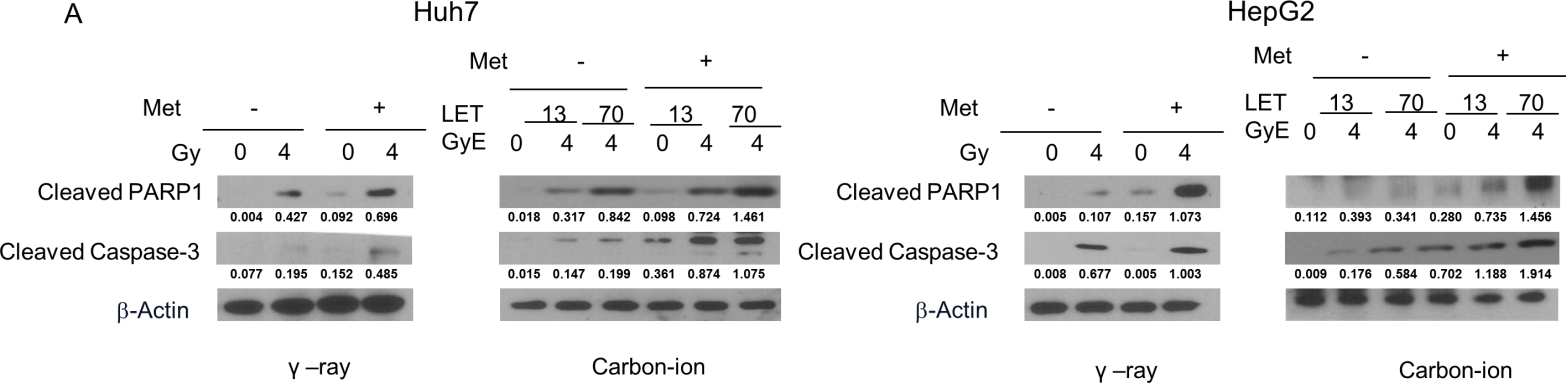
Western blotting analysis of apoptosis-related proteins in Huh7 and HepG2 cells 48 h after γ-rays and carbon ion beams alone or in combination with metformin (Met, 5 mM) β-actin was used as an internal control.

Apoptosis induced by irradiation with or without metformin was investigated by assessing the levels of cleaved caspase-3 and cleaved PARP1 by western blot. As shown in Figure 3, 4 Gy γ-ray or 4 GyE carbon ion beam irradiation induced apoptosis in HCC cells, as indicated by increased cleaved caspase-3 and PARP1. Treatment with metformin alone only slightly increased apoptosis, while combined treatment further increased apoptosis.

**Figure 4:**
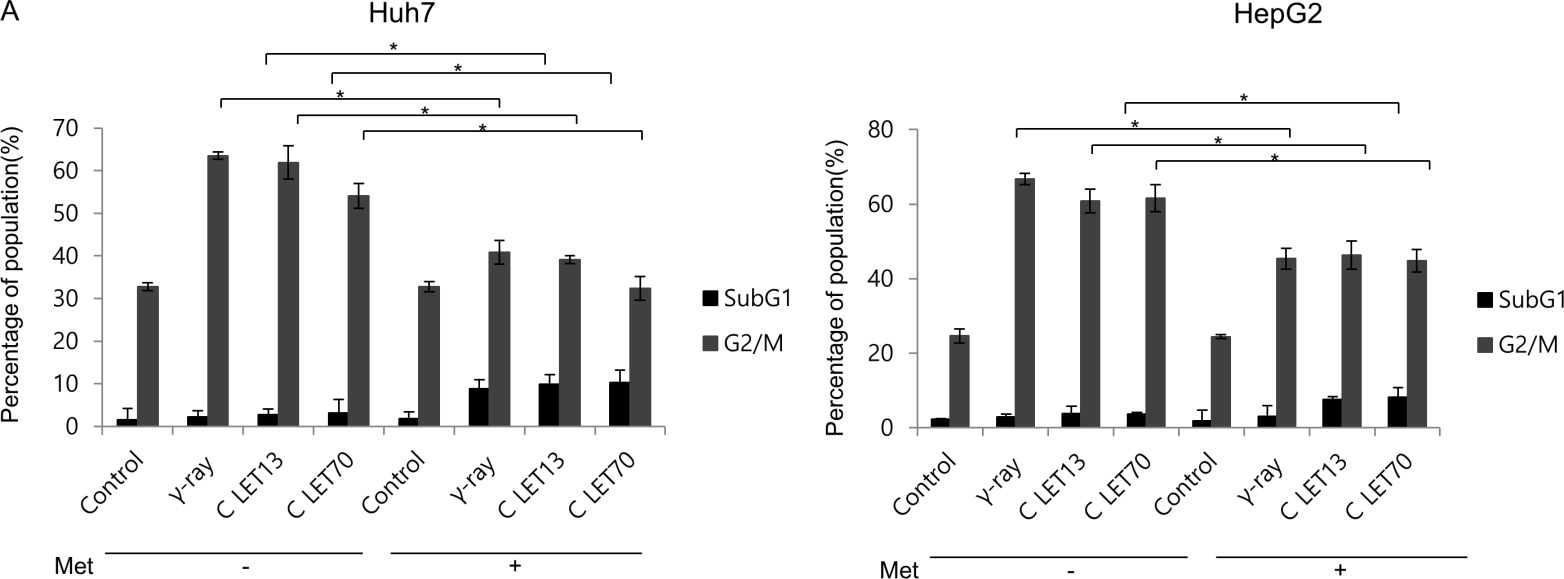
Alteration of cell cycle progression 24 h after γ-ray and carbon ion beams alone or in combination with metformin (Met, 5 mM) Huh7 and HepG2 cells were treated with metformin (Met, 5 mM) and/or 4Gy γ-ray and 4 GyE of carbon ion beams for 24 h. Values represent the means +/− SD of 3 experiments; *p < 0.05, **p < 0.001.

### Changes in cell cycle phase distribution by γ-rays and carbon ion beams alone or in combination with metformin

We next examined the effects of γ-ray and carbon ion beam irradiation alone or in combination with metformin on cell cycle progression using flow cytometry (Figure 4). The most obvious changes in cell cycle distribution following irradiation were increases in G2/M phase cells. Metformin induced G1 arrest and decreased subsequent G2/M arrest after γ-ray, LET 13 carbon-ion, and LET 70 carbon-ion irradiation. Sub-G1 cells, which represent apoptotic cells, were only moderately increased by metformin, but markedly increased by the combined treatment of metformin and γ-ray or carbon ion beam irradiation.

### Changes in tumor cell migration and invasion by γ-rays and carbon ion beams alone or in combination with metformin

**Figure 5:**
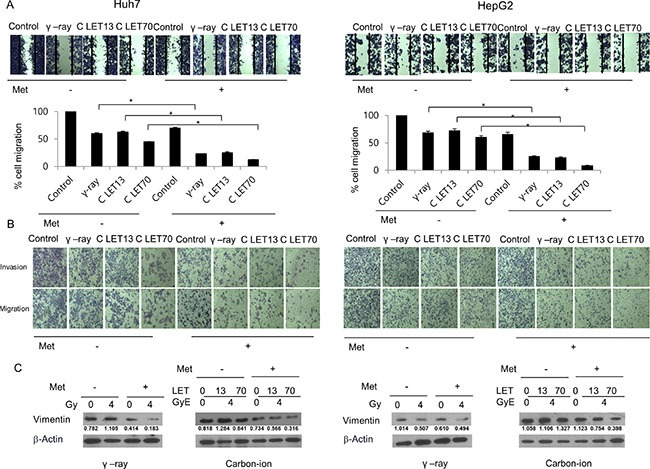
Migration and invasion analysis by a wound-healing scratch assay (A), a transwell chamber assay (B), and western blot (C) after treatment for 24 h Carbon ion beam in combination with metformin (Met, 5 mM) inhibited HCC cell migration and invasion compared with γ-rays.

We next estimated the effects of γ-rays or carbon ion beams alone or combined with metformin on the invasiveness and migration of HCC cells. Irradiation inhibited cell migration toward wound sites, while metformin only slightly inhibited cell migration and invasion (Figure 5A, 5B). However, metformin enhanced the negative effect of γ-rays and carbon ion beams on HCC cell migration. In a Matrigel invasion assay, irradiation with γ-rays or carbon ion beams decreased cell invasiveness, and the combination treatment was more effective in inhibiting tumor cell invasion than irradiation alone, especially with 70keV/μm carbon ion beams. Furthermore, metformin enhanced the effects of γ-rays and carbon ion beams on the decreased expression of vimentin, which is used widely as a marker of the epithelial-mesenchymal transition (EMT) that occurs during metastasis (Figure 5C).

### Activation of the AMPK/mTOR/Akt pathway by γ-rays and carbon ion beams alone or in combination with metformin

**Figure 6:**
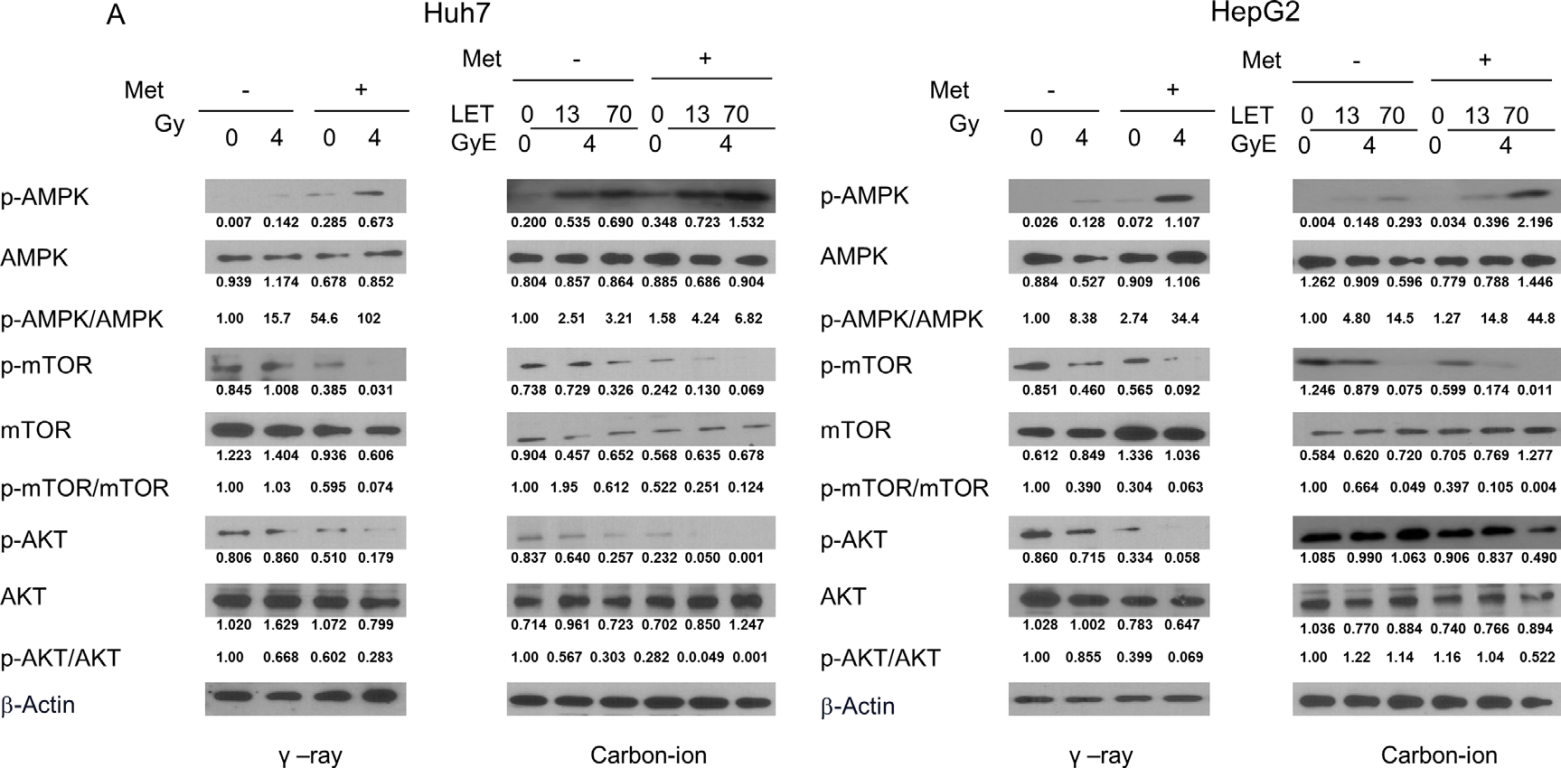
Western blotting analysis of AMPK/mTOR/Akt pathway-related proteins in Huh7 and HepG2 cells 48 h after γ-rays and carbon ion beams alone or in combination with metformin

Finally, we examined the effect of metformin and irradiation alone or in combination on activation of the AMPK/mTOR/Akt signaling pathway using western blots. Treatment of HCC cells for 48 h with 5 mM metformin or irradiation alone increased the expression of p-AMPK and decreased p-mTOR and p-Akt levels. The combined treatment of metformin and irradiation was more effective than metformin or irradiation alone at increasing p-AMPK and decreasing p-mTOR and p-Akt (Figure 6), with the combination of metformin with 70keV/μm carbon ion beams being most effective at altering the expression of these proteins.

## DISCUSSION

Based on cell survival curves, we estimated that the Huh7 (HepG2) RBE value of carbon ion beams with 13 and 70keV/um relative to γ-rays was 1.02 (1.08) and 1.68 (1.75), respectively. LET 13 carbon-ion mono-beams were used because they are representative of the radiation to which normal tissues adjacent to tumors are exposed, while LET 70 beams were used because they are close to the Bragg peak to target tumor volume. With low-LET radiation, including protons and γ-rays, cell survival curves of cancer cells hardly differ between the control group and carbon 13keV/μm LET. However, cells exposed to carbon 70keV/μm LET have differences in cell survival between the Co-60 γ-ray and high-LET groups. In the present study, 70keV/μm carbon ion beam irradiation was more effective than Co-60 γ-ray and 13keV/μm carbon ion beam irradiation in causing clonogenic cell death in HCC cells *in vitro*. Metformin enhanced the effects of ionizing radiation, including Co-60 γ-ray and 13 and 70 keV/μm carbon ion beams to induce clonogenic cell death, apoptosis, γH2AX formation, and inhibition of cell migration in HCC cells.

Metformin is the most frequently used treatment for type II diabetes with few side effects [25–27]. Metformin acts as a chemosensitizer or radiosensitizer in various tumor cell types, including HCC cells [15–19, 25, 26]. In the present study, we first demonstrated that metformin sensitizes HCC cells not only to low-LET photon beams but also to high-LET carbon ion beams. This finding is consistent with previous reports that metformin radiosensitizes HCC cells to high-LET neutron irradiation [24].

Metformin markedly enhanced and also prolonged radiation-induced γH2AX formation such that a significant amount of γH2AX was still present even at 24 h after irradiation. There were no differences in the induction of γH2AX between γ-ray and carbon ion beams alone or in combination with metformin. Metformin also markedly enhanced the DNA damage effects of γ-rays and carbon ion beams and decreased the phospho-DNA-PK expression caused by both γ-ray and carbon ion beams. These findings are consistent with previous reports that the combination of metformin and radiation yielded greater numbers of γH2AX foci than radiation alone, and the reduced phospho-DNA-PKcs is important for metformin to induce radiosensitivity in prostate cancer cells [17, 18]. Taken together, these findings suggest the metformin increases and prolongs DNA damage caused by irradiation with r-rays and high-LET carbon ion beams.

Metformin treatment also enhanced the effects of irradiation on the expression of cleaved caspase-3 and PARP, indicating potentiation of radiation-induced apoptosis in HCC cells. These results are consistent with those of previous reports that metformin potentiated radiation-induced caspase-9/-3 cleavage and PARP-dependent cell death in nasopharyngeal and breast cancer cells [29, 30]. These data may partially explain why carbon ion beams combined with metformin more effectively destroy HCC cells than γ-rays combined with metformin.

AMPK activators and metformin treatment have been shown to inhibit HCC cell proliferation and induce cell cycle arrest at the G1-S checkpoint [30–32]. In this study, metformin alone increased the number of cells in G1 phase, while decreasing the number of cells in G2/M phase. Treatment with γ-ray or carbon ion alone increased the number of cells in G2/M phase and reduced the number of cells in G1 phase. Combined metformin and radiation (γ-ray or carbon ion) treatment reduced G2-M and increased subG1 distribution in comparison to radiation alone, possibly via an enhanced DNA damage response and increased DNA double strand breaks by metformin. Such slowing of cell cycle progression may account for the suppression of cell migration and invasion observed with the combination of metformin and carbon ion beams. The suppression of cell migration and invasion by metformin suggests that metformin may reduce metastasis in clinical settings. Because the radiosensitizing effects of metformin are intended to enhance tumor cell death, experiments with mouse models should be conducted first to examine the possible complications in clinical applications.

We also observed that the combination of metformin with γ-rays and carbon beams was more effective than γ-rays or carbon ion beams alone in increasing the expression of p-AMPK and suppressing the expression of p-mTOR. This finding is consistent with recent reports that the AMPK/mTOR pathway may be the target of the anti-proliferative effects of metformin in combination with ionizing radiation in esophageal and gastric cancer cells [33–36]. Other recent reports have also shown that metformin inhibits the expression of N-cadherin, MMP-2, and MMP-9 proteins in an AMPK/p53-dependent manner in melanoma and suppresses MMP-9 in HCC cells [37, 38].

Importantly, it has been recently reported that metformin is cytotoxic to cancer stem cells (CSCs) and enhances the effects of chemotherapy and radiation against CSCs in many types of human cancer, including HCC, glioblastoma, breast, pancreas, lung, and esophageal cancer [31, 35,39–44]. Based on our previous observation that carbon ion beams are effective in killing CSCs [15, 45, 46], in future studies we plan to elucidate the combination of metformin and carbon ion beams against CSCs.

In conclusion, metformin sensitized HCC cells to γ-ray irradiation and carbon ion beams, as indicated by the increase in clonogenic cell death, DNA damage, apoptosis, and cell cycle arrest and the inhibition of tumor cell migration and invasion. Enhanced activation of AMPK and down-regulation of mTOR and Akt appeared to play a role in the metformin-induced sensitization of cancer cells to γ-rays and carbon ion beams.

## MATERIALS AND METHODS

### Antibodies and chemicals

Anti-phospho-DNA-PK, anti-cleaved PARP1, anti-cleaved caspase-3, anti-phospho-AMPK, anti-AMPK, anti-phospho-mTOR, anti-mTOR, anti-phospho-AKT and anti-AKT were all purchased from Cell Signaling Technology (Danvers, MA, USA). Anti-vimentin and anti-b-Actin were purchased from Santa Cruz Biotechnology (Santa Cruz, CA, USA). Anti-γ H2AX was obtained from Millipore (Billerica, MA, USA). Metformin (1-(diaminomethylidene)-3, 3-dimethylguanidine) was purchased from Sigma-Aldrich Chemical Corp (St. Louis, MO, USA).

### Cell culture

The HCC cell lines Huh7 and HepG2 were purchased from Japanese Collection of Research Bioresources (JCRB, Tokyo, Japan) and cultured in Dulbecco's minimal essential medium (DMEM; GIBCO, Gaithersburg, MD, USA) supplemented with heat-inactivated 10% fetal bovine serum (FBS; GIBCO), 0.1 mM non-essential amino acids, glutamine, 4-(2-hydroxyethyl)-1-piperazineethanesulfonic acid (HEPES), and antibiotics at 37°C in a 5% CO_2_ humidified incubator.

### Irradiation

Cells were plated in 60-mm plastic dishes and incubated at 37°C under a humidified 5% CO_2_ atmosphere. Cells at 70–80% confluence were irradiated with a ^137^Cs γ-ray source (Atomic Energy of Canada, Ltd., Ontario, Canada) at a dose rate of 3.81 Gy/min. Carbon ions were provided by 290 MeV/nucleon beams at NIRS-HIMAC. Cells were irradiated with mono-peak C-ions with a dose-averaged LET of 13 or 70keV/μm at a dose rate of 2.0–5.0 Gy/min. The dose unit in the literature was expressed as Gray equivalent (GyE), which is determined for the clinical situation as previously described [47].

### Colony-forming assay

Cells were seeded into 6-well plates in triplicate and irradiated with or without metformin. After incubation for 14–20 days, colonies were stained with 0.4% crystal violet (Sigma, St. Louis, MO, USA), and the plating efficiency (PE) was determined. The PE of Huh7 and HepG2 were 0.48 ± 0.18, 0.45 ± 0.15, respectively. The surviving cell fraction was calculated as follows: surviving fraction = colonies counted/(cells seeded × PE/100). The radiosensitizing effect of metformin was calculated by dividing the radiation dose (Gy) for radiation alone by the dose for radiation plus metformin at a surviving fraction of 10%.

### Flow cytometry

Cells were cultured, harvested at the indicated times, stained with propidium iodide (1 μg/ml, Sigma) according to the manufacturer's protocol, and then analyzed using a FACScan flow cytometer (Becton Dickinson, Franklin Lakes, NJ, USA). A minimum of 10,000 cells was counted for each sample, and data analysis was performed using CellQuest software (BD Biosciences).

### Immunocytochemistry

γH2AX expression was assessed using an immunocytochemistry assay method [46]. Cells were grown on chambered slides for 1 day, pretreated with metformin for 6 or 24 h, and then irradiated. Cells were then fixed with 4% paraformaldehyde and permeabilized with 0.5% Triton X-100 in PBS. Detection was performed after the slides were blocked in 4% FBS in PBS for 1 h and stained with a 1:100 dilution of a primary antibody against γ-H2AX (Millipore, Billerica, MA, USA) and a 1:500 dilution of FITC-labeled secondary antibodies.

### Western blotting

HCC cells in the exponential growth phase were pretreated with metformin for 8 h and then irradiated for 6 or 24 h. The cells were then lysed with RIPA buffer, and proteins were separated by SDS-polyacrylamide gel electrophoresis (PAGE) and transferred to nitrocellulose membranes. The membranes were blocked with 1% (v/v) nonfat dry milk in Tris-buffered saline with 0.05% Tween20 and incubated with antibodies. Blots were reacted with primary antibodies at 1:1000 dilution and secondary antibodies at 1:5000 dilution. Immunoreactive protein bands were visualized by Enhanced Chemi-luminescence (Amersham Biosciences) and scanned.

### Neutral comet assay

The radiation-induced DNA double-strand breaks were determined by the comet assay (single cell gel electrophoresis assay) as previously described [24]. Cells were plated in 100-mm tissue culture dishes at 1 × 10^6^/dish and incubated overnight. After metformin exposure for 8 h, the cells were irradiated and incubated for 6 or 24 h, and a neutral comet assay was performed using a comet assay kit (Trevigen, Gaithersburg, MD, USA). The cells were lysed at 4°C for 1 h in lysis buffer (2.5 M NaCl, 100 mM ethylenediaminetetraacetic acid, 10 mM Tris-HCl, 1% *N*-lauroylsarcosine, pH 7) and subjected to neutral electrophoresis at 4°C. The slides were stained with ethidium bromide and examined for fluorescence emission using an excitation filter of 515–560 nm and a barrier filter of 590 nm. DNA damage was assessed via computer-assisted image analysis (Komet analysis software, ver. 3.1; Kinetic Imaging, Liverpool, UK).

### Wound-healing scratch assay

Cells were seeded onto six-well plates (Corning) at 2.5 × 10^4^ cells/well with 3 ml of DMEM with 10% FBS. After metformin pre-incubation for 8 h, the cells were irradiated. On day 2, the monolayers were mechanically disrupted with a sterile 200-μl pipette tip. The assay was performed in duplicate. Wells were imaged after incubation for 24 h, and cell migration was measured using a Nikon Eclipse Ti microscope with a DS-Fi1 camera. Cells were counted using ImageJ software (United States National Institutes of Health, Bethesda, MD, USA).

### Invasion assay

The invasive ability of cells was measured using a transwell invasion assay kit (Chemicon, Millipore, GA, USA), according to the manufacturer's protocol. Briefly, cells were seeded onto the membrane of the upper chamber of the transwell at a concentration of 4 × 10^5^ cells/ml in 150 μl of serum-free RPMI and were left untreated or treated with the indicated doses of metformin, radiation, or a combination of both. The medium in the lower chamber contained 10% FBS as a chemoattractant. After incubation for 24 h, cells that passed through the Matrigel-coated membrane were stained with the Cell Stain Solution containing crystal violet and imaged.

### Statistical analyses

All analyses were determined by Student's *t*-test. Differences were considered significant if the *p* value was less than 0.05 or 0.001.
